# Giant Abdominal Aortic Aneurysm in Bone Scan

**DOI:** 10.4274/mirt.galenos.2018.55477

**Published:** 2019-06-24

**Authors:** Derya Çayır, Mehmet Bozkurt, Özdeş Emer, Salih Sinan Gültekin, Alper Özgür Karacalıoğlu

**Affiliations:** 1 University of Health Sciences, Dışkapı Yıldırım Beyazıt Training and Research Hospital, Clinic of Nuclear Medicine, Ankara, Turkey; 2 University of Health Sciences, Gülhane Training and Research Hospital, Clinic of Nuclear Medicine, Ankara, Turkey

**Keywords:** Whole body scan, Tc-99m methylene diphosphonate, abdominal aortic aneurysm, metastases, PET/CT

## Abstract

Abdominal aortic aneurysm (AAA) may be incidentally detected in three-phased bone scintigraphy. AAA should be diagnosed prior to the development of symptoms to perform elective repair surgery. We present a rare case who presented with back pain and underwent a 3-phase bone scan with Tc-99m methylene diphosphonate, which revealed a giant AAA on blood-flow and blood-pool phases in addition to bone metastases. F-18-fluorodeoxyglucose positron emission tomography/computed tomography (CT) identified hypermetabolic liver, lung, and bone lesions, and CT component of the study confirmed the diagnosis of AAA with a maximum diameter of 92 mm. The initial two phases of a 3-phase bone scintigraphy are decisive to identify vascular pathologies that may be life-threatening, if left untreated.

## Abstract (tr)

Abdominal aort anevrizması (AAA), 3-fazlı kemik sintigrafisinde insidental olarak saptanabilmektedir. Semptomlar ortaya çıkmadan elektif cerrahi uygulanabilmesi için, AAA tanısının doğru olarak konulması önemlidir. Burada, sırt ağrısı şikayeti ile başvuran bir olguda, Tc-99m metilen difosfonat 3-fazlı kemik sintigrafisinde kemik metastazlarının yanı sıra kan akımı ve kan havuzu fazlarında dev AAA saptanan nadir bir olgu sunuyoruz. F-18 florodeoksiglukoz pozitron emisyon tomografi/bilgisayarlı tomografide (BT) hipermetabolik karaciğer, akciğer ve kemik lezyonları görüldü ve çalışmanın BT bileşeninde maksimum çapı 92 mm olan AAA tanısı doğrulandı. 3-fazlı kemik sintigrafisinde, tedavi edilmediği takdirde yaşamı tehdit edebilecek vasküler patolojileri tanımlamak için ilk iki faz belirleyicidir.

## Figures and Tables

**Figure 1:**
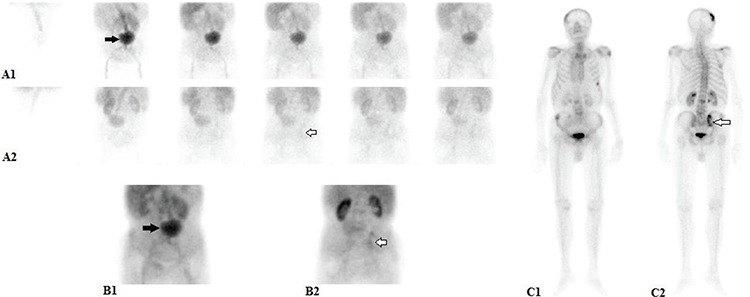
An 84-year-old man presented with low back pain since two months. Lumbar magnetic resonance imaging revealed hypointense lesions in the vertebral column on T1- and T2-weighted images. The patient was referred to 3-phase bone scan for evaluation of suspected bone metastasis of unknown origin. Dynamic blood-flow and static blood-pool images were obtained following intravenous bolus injection of 740 MBq (20 mCi) Tc-99m methylene diphosphonate. Blood-flow and blood-pool phase images demonstrated tracer accumulation in the left side of the mid-abdominal portion of the infrarenal area (blood-flow phase, anterior: A1; posterior: A2, black arrows), and tracer activity consistent with hyperemia in the right sacroiliac joint (blood-pool phase, anterior: B1; posterior: B2, white arrows). Late phase images showed abnormal tracer uptakes in the right parietal bone of the skull, right scapular spine, anterior side of the left 6^th^ costa, posterior aspect of the left 12^th^ costa, L2 and L5 vertebrae, as well as the right sacroiliac joint (late phase, anterior: C1; posterior: C2, white arrow)

**Figure:**
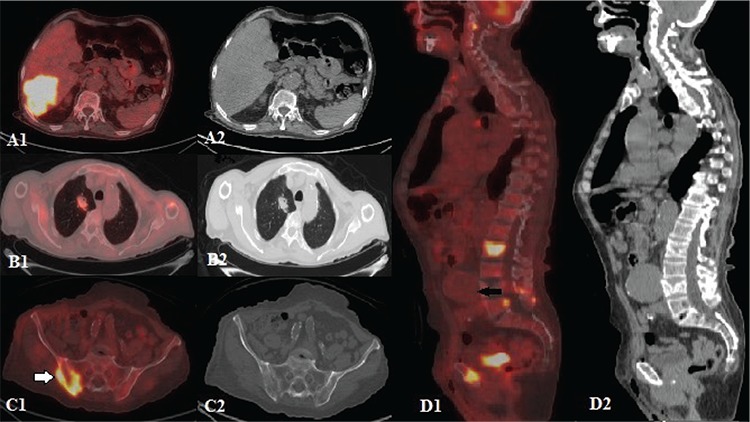
An F-18-fluorodeoxyglucose positron emission tomography/computed tomography was performed to identify the primary tumor site and revealed a mass lesion (86x74x120 mm) in the right lobe of the liver (SUV_max_: 21.2) (A1, A2), an irregularly contoured right lung upper lobe anterior segment mass (27x35 mm, SUV_max_: 4.6) (B1, B2), right hilar and subcarinal lymph nodes, and bone lesions in right iliac crest (SUV_max_: 14.5) (C1, C2, white arrow) which were hypermetabolic, along with a hypometabolic giant abdominal aortic aneurysm (AAA) (65x92x79 mm) (D1, D2, black arrow). The patient did not have a history of trauma or infection, therefore the lesion was diagnosed as a true aneurysm. The patient was referred to cardiovascular surgery for surgical intervention and interventional radiology for liver biopsy AAA is dilation of the abdominal aorta greater than 50% of the normal aortic diameter (1). For most adults, an infrarenal aorta with a maximum diameter of ≥3.0 cm is considered an aneurysm (1,2,3). AAA is more likely found among men, and only 1-2% of male patients are older than 50 years (4,5). More than 90% of patients with AAA are current or past smokers, and smoking is more closely associated with AAA than atherosclerotic diseases (6). AAA should be identified accurately prior to development of symptoms and elective repair is the mainstay of treatment to prevent rupture and sudden death, especially for patients who have AAA with a maximum diameter >5.5 cm, a saccular aneurysm, or an abdominal or back pain that can be attributable to AAA. Immediate repair is recommended for patients who present with a ruptured aneurysm (1). In three-phase bone scans, vascular pathologies (AAA, iliofemoral occlusive arterial abnormalities, and lower extremity varicose veins) that could not be detected in conventional bone scintigraphy, may be detected incidentally in blood-flow and blood-pool phases, depending on lesion vascularity (7). False AAAs in three-phase bone scan has been reported previously (8,9). However, to the best of our knowledge, our case is the first reported true AAA that was shown in a three-phase bone scan.
